# Investigating the Association between Cognitive Function with Retinal Thickness, Choroidal Thickness and Lens Opacities in People with and without Down Syndrome

**DOI:** 10.1002/alz70856_103254

**Published:** 2025-12-25

**Authors:** Aoife M.L. Hunter, Sarah Atkinson, Elaine Murray, Kathryn Saunders, Lajos Csincsik, Jamie Mitchell, Tunde Peto, Julie‐Anne Little, Imre Lengyel

**Affiliations:** ^1^ Ulster University, Coleraine, United Kingdom; ^2^ Queen's University Belfast, Belfast, United Kingdom

## Abstract

**Background:**

The risk of Alzheimer's disease (AD) is substantially higher in Down Syndrome (DS), making early detection of those at risk of AD crucial. This study investigated the association between cognitive function with peripapillary retinal nerve fibre layer thickness (pRNFLT), whole retinal thickness (WRT), choroidal thickness (CT) and small dot lens opacities (SDLO) in DS and healthy controls (HC).

**Method:**

pRNFLT (scan diameter: 3.4mm), WRT (horizontal posterior pole scan size: 30°x25°) and CT (Enhanced Depth Imaging foveal 30° scan) were measured in one eye using Spectral Domain Optical Coherence Tomography (SD‐OCT, Heidelberg Spectralis) in 50 HC (mean age: 66.2 years, range: 50‐80) and 15 DS participants (mean age: 20.9 years, range: 7‐36). Slit‐lamp (SL) photography (Nikon FS‐3 SL) was used to image the crystalline lens, and images graded based on presence or absence of SDLO. Cognitive abilities (composite IQ scores) were assessed using Kaufman Brief Intelligence Test 2 (KBIT‐2). Multivariate linear regression models assessed relationships between thickness measures (response variables: pRNFLT [global, nasal, superonasal, inferonasal, temporal, superotemporal, and inferotemporal sectors], WRT [ETDRS grid sectors] and subfoveal CT) and both composite IQ and SDLO presence (fixed effects), with group (DS/HC) as a covariate. Age and axial length were included as fixed effects.

**Result:**

Positive significant relationships were observed between IQ and global (Beta coefficient [β]=0.28, *p* = 0.040), inferonasal (β=0.60, *p* = 0.028), and inferotemporal pRNFLT (β=0.50, *p* = 0.049). Positive significant relationships were observed between SDLO presence (HC=0%, DS=40%) and inferotemporal (β=45.8, *p* = 0.0018) and temporal pRNFLT (β=18.1, *p* = 0.036). Superonasal pRNFLT was negatively associated with SDLO presence (β=‐31.3, *p* = 0.021). No significant associations existed between IQ and global, inner and outer circle WRT ETDRS grid sectors (global: β=0.11, *p* = 0.50; inner: β=0.14, *p* = 0.44; outer: β=0.12, *p* = 0.47) or SDLO presence (global: β=11.9, *p* = 0.14; inner: β=15.7 *p* = 0.08; outer: β=6.68, *p* = 0.40). Subfoveal CT was not significantly associated with IQ (β=0.12, *p* = 0.91) or SDLO presence (β=‐41.85, *p* = 0.40), but had a positive association with DS (β=171.1, *p* = 0.0499).

**Conclusion:**

HC and DS participants with thicker pRNFL exhibited better cognitive abilities. DS participants with SDLO exhibited thicker inferotemporal and temporal, but thinner superonasal pRNFL. The pRNFL may be a valuable marker of cognitive function in people with and without DS.

